# Supplementary material for: A potent nonapeptide inhibitor for the CXCL12/HMGB1 heterocomplex: A computational and experimental approach
Source: Comput Struct Biotechnol J. 2025 Apr 18;27:1677–85. doi: 10.1016/j.csbj.2025.04.023 (PMC12925725; doi:10.1016/j.csbj.2025.04.023)
Supplement: Supplementary file 1 — Supplementary material [file mmc1.pdf]

Supplementary Materials

A Potent Nonapeptide Inhibitor for the CXCL12/HMGB1 heterocomplex: A Computational and Experimental Approach

Enrico Mario Alessandro Fassi[1,#], Edisa Pirani[2,#], Valentina Cecchinato[2], Andrea Cavalli[2,3], Gabriella Roda[1], Mariagrazia Uguccioni[2,§,*], Jacopo Sgrignani[2,§,*], Giovanni Grazioso[1,§,*]

[1] Department of Pharmaceutical Sciences, Università degli Studi di Milano, Via L. Mangiagalli 25, 20133 Milano, Italy.

[2] Institute for Research in Biomedicine (IRB), Università della Svizzera italiana, Via Chiesa 5, 6500 Bellinzona, Switzerland.

[3] Swiss Institute of Bioinformatics (SIB), University of Lausanne, Quartier UNIL-Sorge, Bâtiment Amphipôle, 1015 Lausanne, Switzerland.

[#] These authors equally contributed.

* Joint senior and corresponding authors: giovanni.grazioso@unimi.it; jacopo.sgrignani@irb.usi.ch; mariagrazia.uguccioni@irb.usi.ch

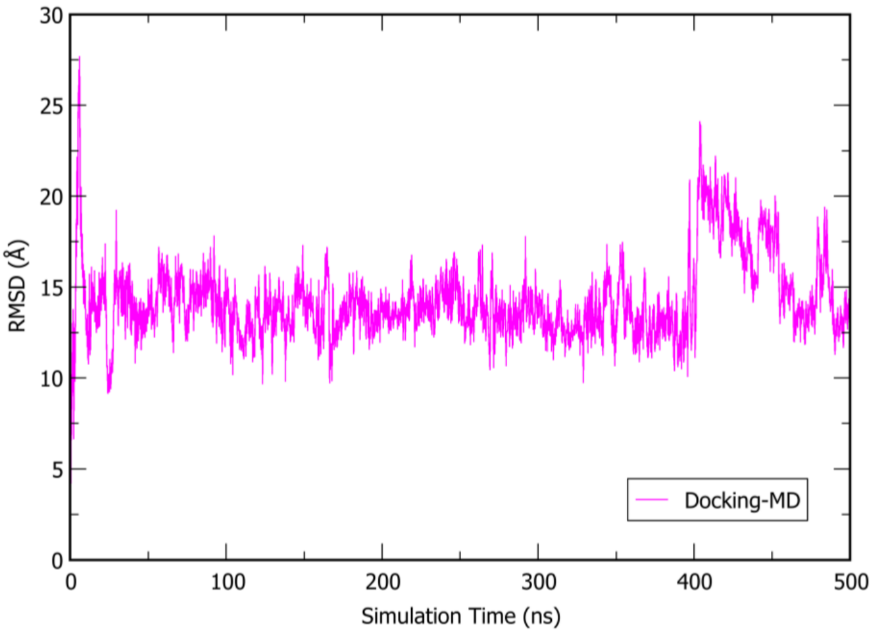

Figure S1. RMSD analysis of the HBP08/HMGB1-BoxB complex model subjected to 500 ns-long MD simulation. The complex structure was retrieved in our previous work published (DOI: 10.1021/acs.jmedchem.1c00852), that was obtained by computational docking guided by the protein residues involved in the peptide binding as reported by NMR CSP experiments performed.

Table S1. Binding free energy (ΔG) values of each independent replica of the HBP08/HMGB1-BoxB complexes.

HBP08	ΔG ± SE (kcal/mol)
MD replica1	-31.9 ± 0.4
MD replica2	-31.2 ± 0.3
MD replica3	-36.4 ± 0.5

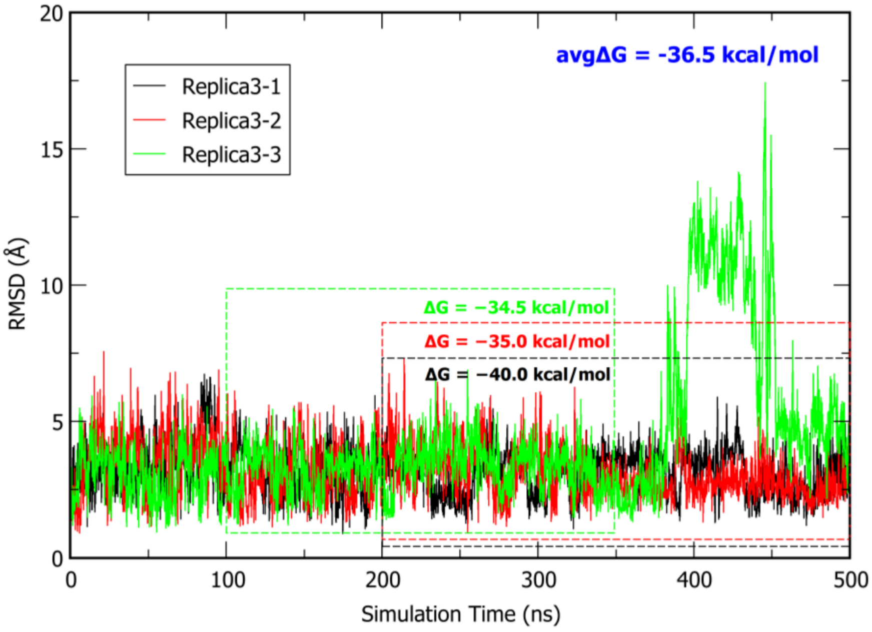

Figure S2. RMSD analysis of replica3-1 (black), replica3-2 (red) and replica3-3 (green) of HBP08 in complex with HMGB1-BoxB protein. In broken lines are highlighted the snapshots considered in the MM-GBSA calculations. The ΔG values obtained for each replica and the average value are reported.

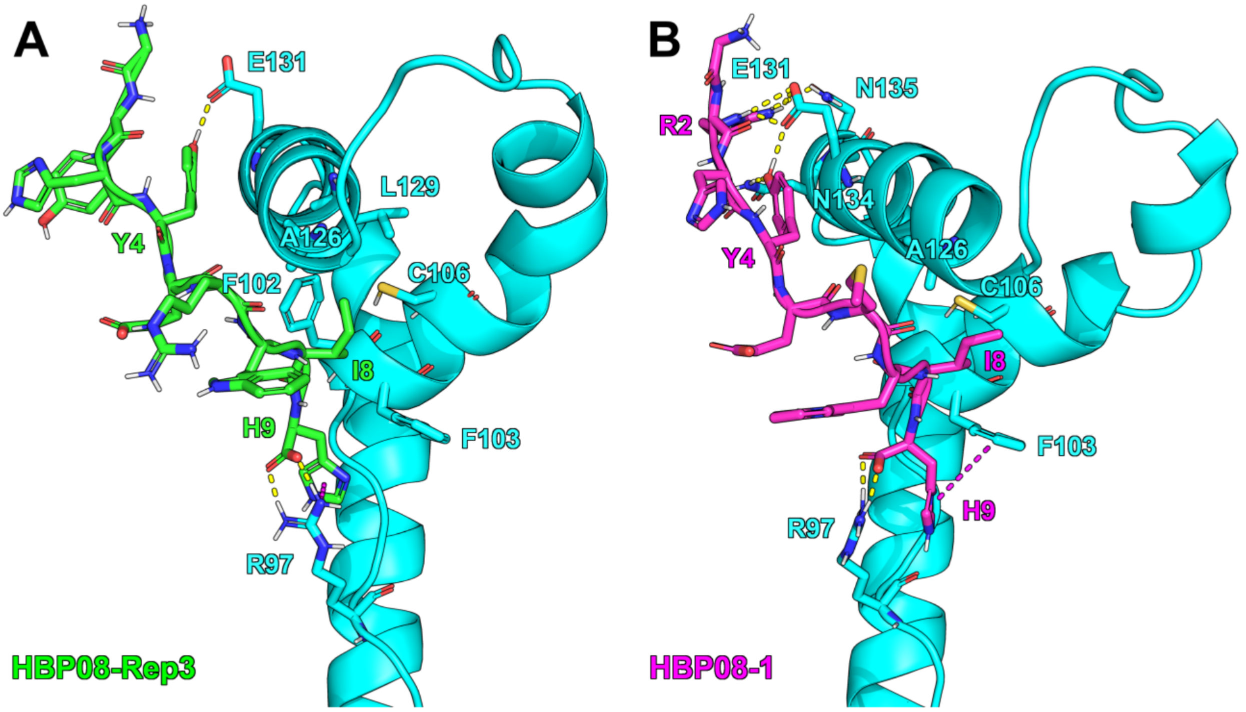

Figure S3. Representative structure of the most populated cluster of (A) HBP08-Replica3 (with sequence GYHYERWIH, green sticks) and (B) HBP08-1 peptide (with sequence GRHYEMWIH, magenta sticks) in complex with HMGB1-BoxB, considering 1.5 µs of MD simulations. These complexes were used as starting complex structure for the following affinity maturation steps. H-bond and π-π interactions are represented as yellow and purple dashed lines, respectively.

Table S2. Binding free energy (ΔG) of the mutated peptide HBP08-1 compared to the parent peptide HBP08.

Peptide	Sequence	ΔG Rep1 ± SE [1]	ΔG Rep2 ± SE [1]	ΔG Rep3 ± SE [1]	Average ΔG [1]
HBP08	GYHYERWIH	-40.0 ± 0.2	-35.0 ± 0.3	-34.5 ± 0.2	-36.5
HBP08-1	G**R**HYE**M**WIH	-39.5 ± 0.3	-39.5 ± 0.3	-34.9 ± 0.2	-38.0

[1] (kcal/mol).

Table S3. Binding free energy (ΔG) of the HBP08-1 mutated peptides in complex with HMGB1-BoxB, derived from the affinity maturation protocol in which H3 and I8 were simultaneously mutated, subjected to 500 ns MD simulations.

Group	Mutation	Sequence	ΔAffinity [1]	ΔStability [1]	ΔG ± SE [1]
**HBP08-1**	/	G**R**HYE**M**WIH	/	/	−38.0
**Affinity**	H3E + I8M	GREYEMWMH	−12.19	−2.26	−37.5 ± 0.3
	H3Y + I8M	GRYYEMWMH	−11.64	0.00	−35.4 ± 0.3
	H3N + I8M	GRNYEMWMH	−9.92	+4.08	−36.5 ± 0.2
**Stability**	H3R + I8L	GRRYEMWLH	+3.09	−13.56	−35.9 ± 0.3
	H3R + I8G	GRRYEMWGH	+19.01	−13.35	*unbound*
	H3R + I8M	GRRYEMWMH	−7.32	−13.14	−37.5 ± 0.2
**Mixed**	H3Q + I8M	GRQYEMWMH	−8.88	−4.54	−34.2 ± 0.3
	H3L + I8M	GRLYEMWMH	−8.39	−5.44	−32.8 ± 0.3

[1] (kcal/mol).

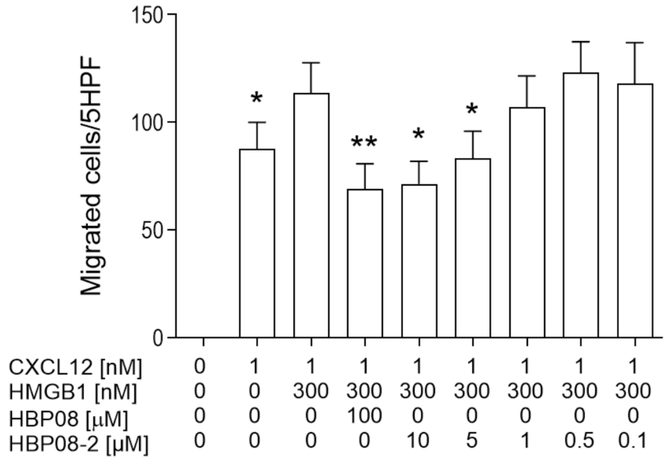

Figure S4. *In vitro* inhibition of CXCR4-transfected 300-19 Pre-B cells in response to the CXCL12/HMGB1 heterocomplex using the HBP08-2 peptide. (A) Inhibition of cell migration in response to the CXCL12/HMGB1 heterocomplex was assessed on 300-19 Pre-B cells CXCR4-transfected using the identified peptide HBP08-2 or HBP08 (as a control). Data are shown as the mean + SEM of four independent experiments performed. *$p < 0.05$; **$p < 0.01$; by one-way ANOVA, followed by Dunnett's multicomparison test, comparing each condition to the migration observed in response to the CXCL12/HMGB1 heterocomplex.

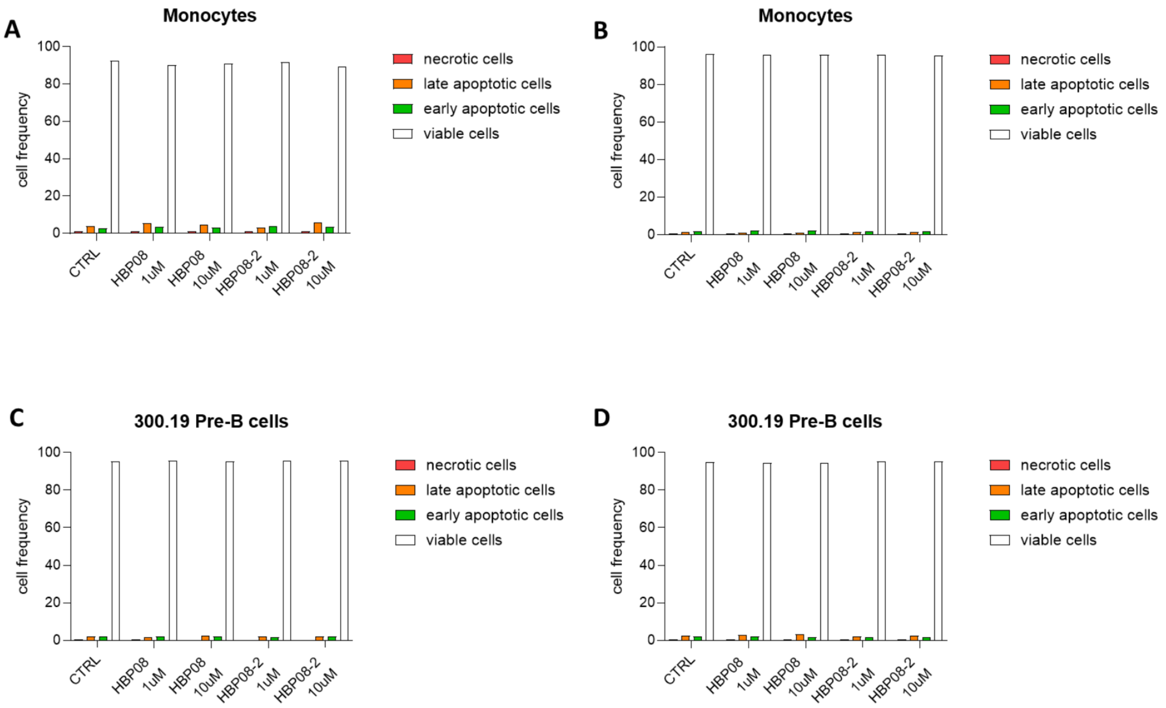

Figure S5. *In vitro* determination of cell viability by Annexin V/PI staining and flow cytometric analysis. Monocytes (A-B) and 300.19 Pre-B cells transfected with CXCR4 (C-D) were treated with 1 or 10 µM of HBP08 or HBP08-2 for 2 hours (A-C) or 4 hours (B-D).

Table S4. Dataset overview of the Microscale Thermophoresis (MST) experiments accomplished using a fixed 10 nM concentration of target proteins (HMGB1-BoxB, HMGB1-BoxA and full sequence HMGB1) and different concentrations of HBP08-2 and HBP08-3 peptides. At least two independent experiments were performed to compute the K_d value.

Peptide	Target Protein	MST Power	Exc. Power	Temp.	[Ligand] Range	Time	RA	SNR	K_d (nM)
HBP08-2	HMGB1-BoxB	40%	20%	25 °C	25 µM – 0.763 nM	5 s	17.8	20.1	11.3 ± 2.3
HBP08-3	HMGB1-BoxB	40%	20%	25 °C	25 µM – 0.763 nM	5 s	17.4	31.9	15.3 ± 1.9
HBP08-2	HMGB1-BoxA	40%	20%	25 °C	78.10 µM – 9.54 nM	5 s	21.4	36.6	4243 ± 447
HBP08-2	HMGB1	40%	20%	25 °C	6.25 µM – 0.19 nM	5 s	18.1	14.2	28.1 ± 7.0

RA = Response Amplitude; SNR = Signal-to-Noise Ratio.

Sample:	HBP08-2
Lot. No.:	P201104-HS843561
Column:	Gemini-NX 5μ C18 110A, 4.6*250mm
Solvent A	A: 0.1% Trifluoroacetic Acid in 100% Acetonitrile
Solvent B	B: 0.1% Trifluoroacetic Acid in 100% Water

Gradient:

	A	B
0.0min	15%	85%
25.0min	40%	60%
25.1min	100%	0%
30.0min	Stop	

Volume:	10μl
Wavelength:	220nm
Flow rate:	1.0ml/min

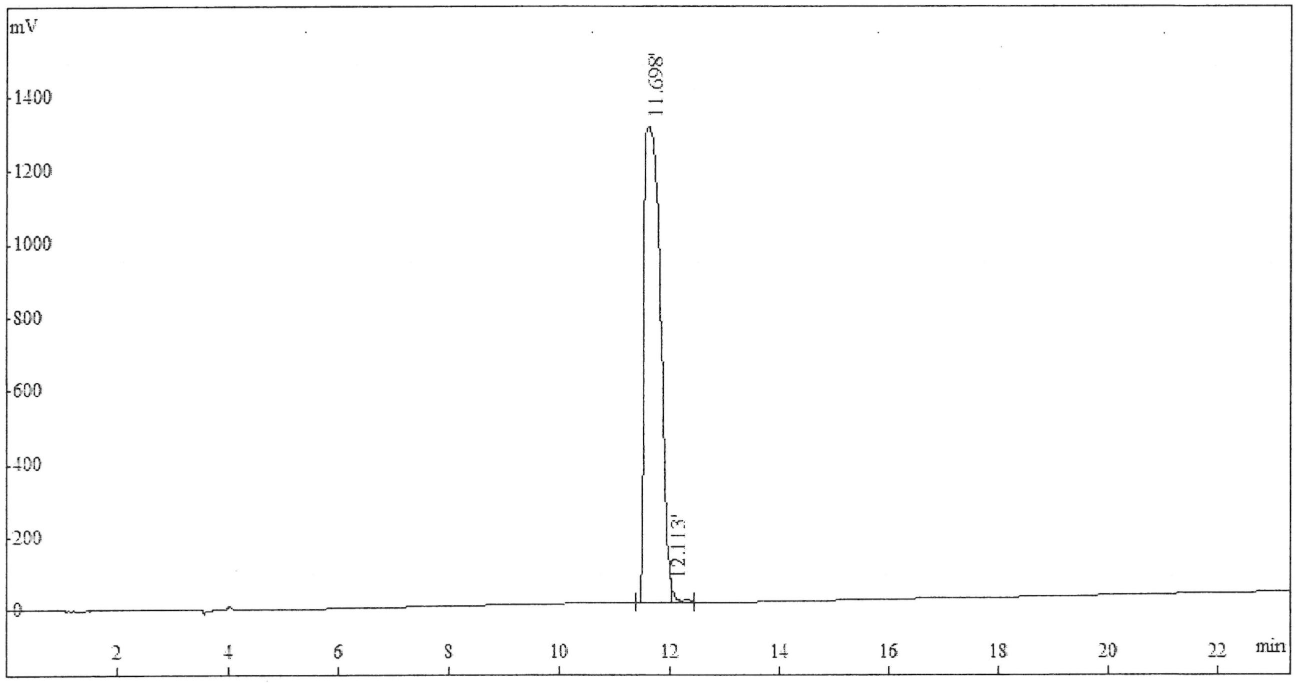

Rank	Time	Conc.	Area	Height
1	11.698	98.9953	25096146	1302599
2	12.113	1.0047	254704	40496
Total		100	25350850	1343095

MASS SPECTROMETRY REPORT

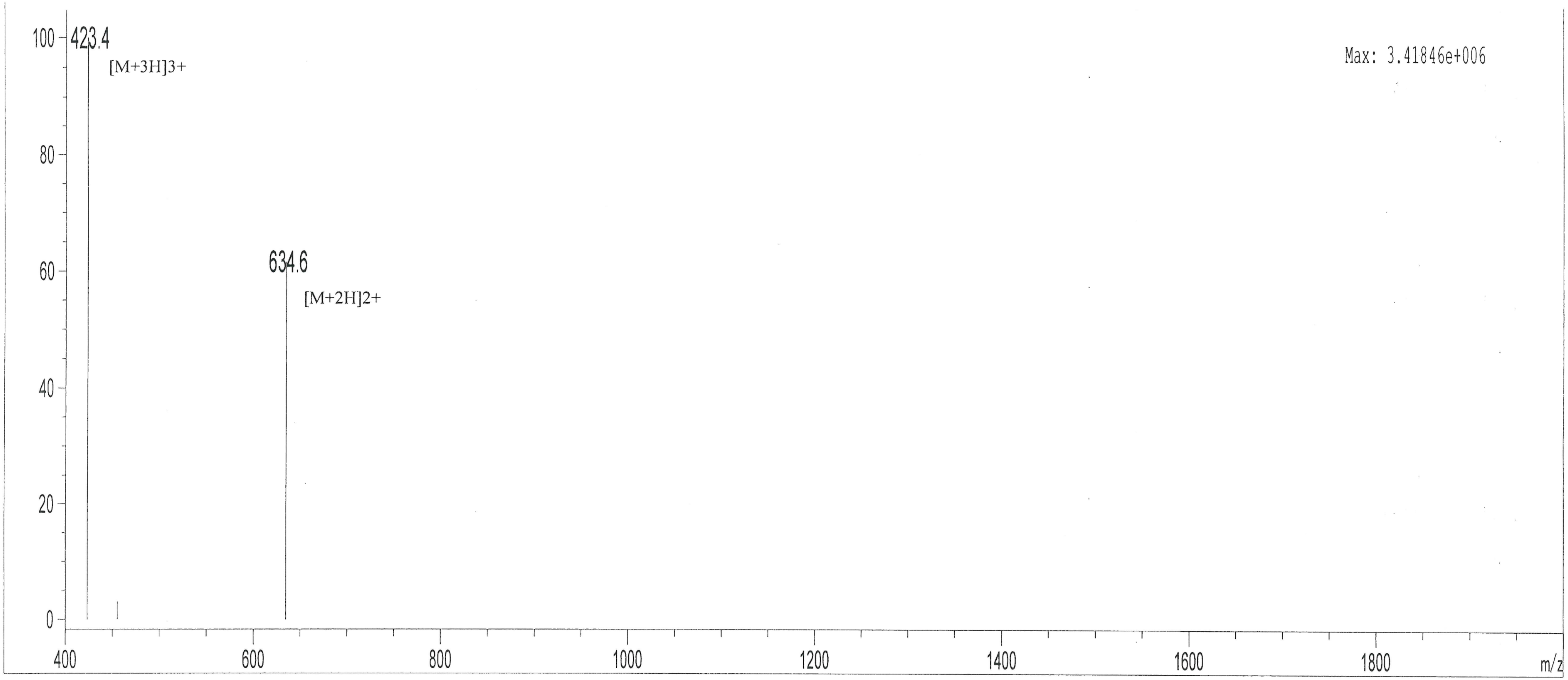

Sample Information		Probe:	ESI	Probe Bias:	+4.5kv
Injection Volume	: 1.00 μ l	Nebulizer Gas Flow:	1.5L/min	Detector:	1.5kv
Sample:	HBP08-2	CDL:	-20.0v	T. Flow:	0.2ml/min
M.W.:	1267.46	CDL Temp.:	250 °C	B. Conc.:	50%H2O/50%ACN
Lot. No.:	P201104-HS843561	Block Temp.:	200 °C		

Sample:	HBP08-3		
Lot. No.:	P201104-HS843560		
Column:	Gemini-NX 5μ C18 110A, 4.6*250mm		
Solvent A	A: 0.1% Trifluoroacetic Acid in 100% Acetonitrile		
Solvent B	B: 0.1% Trifluoroacetic Acid in 100% Water		
Gradient:		A	B
	0.0min	20%	80%
	25.0min	45%	55%
	25.1min	100%	0%
	30.0min	Stop	
Volume:	10μl		
Wavelength:	220nm		
Flow rate:	1.0ml/min		

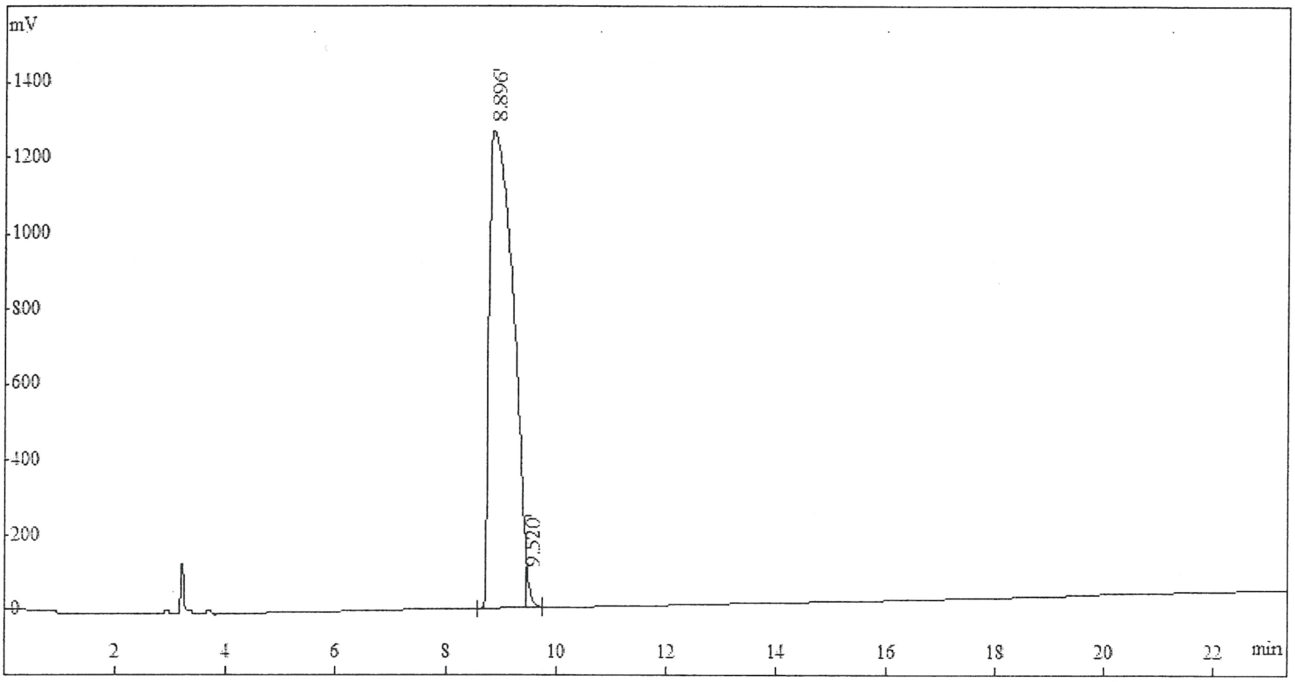

Rank	Time	Conc.	Area	Height
1	8.896	99.0767	37340397	1273107
2	9.520	0.9233	347977	84758
Total		100	37688374	1357865

MASS SPECTROMETRY REPORT

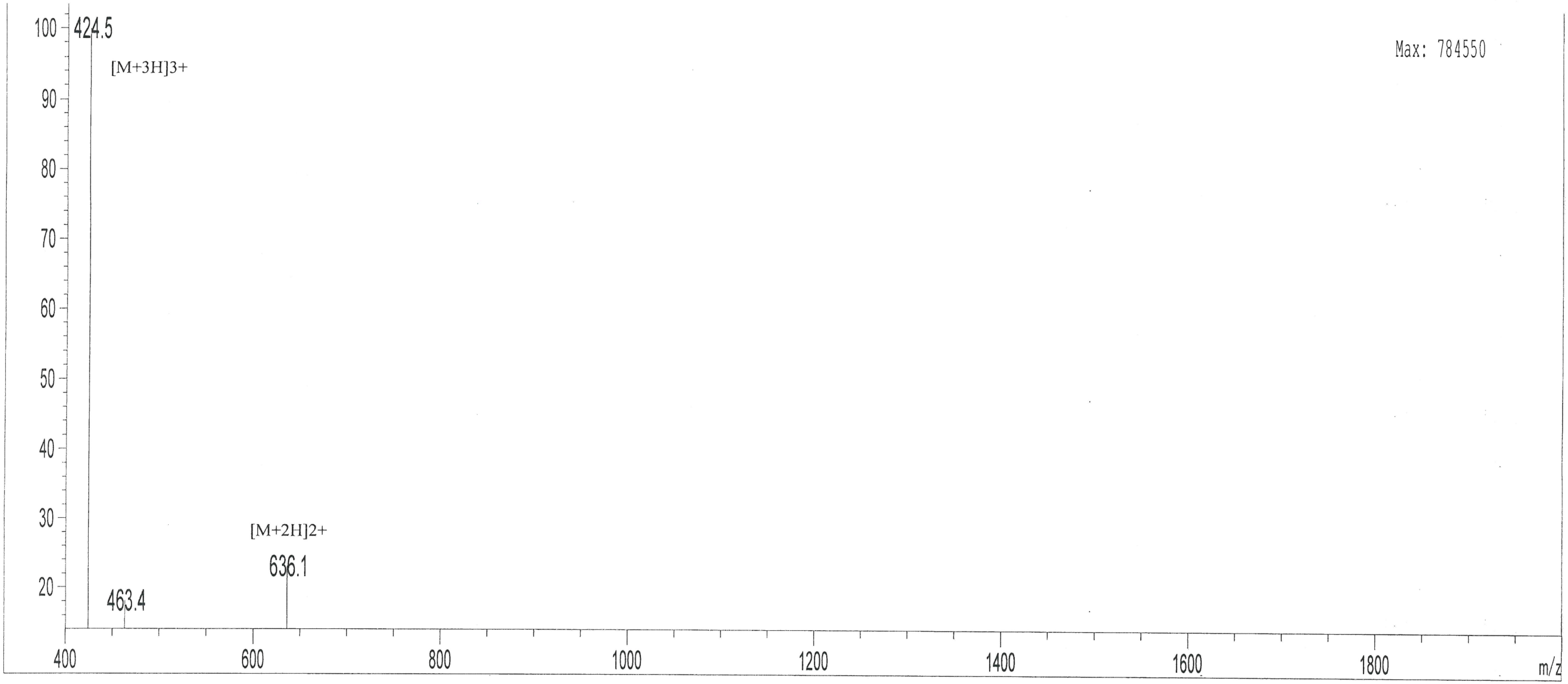

Sample Information

Injection Volume	: 1.00 μ l	Probe:	ESI	Probe Bias:	+4.5kv
Sample:	HBP08-3	Nebulizer Gas Flow:	1.5L/min	Detector:	1.5kv
M.W.:	1270.52	CDL:	-20.0v	T. Flow:	0.2ml/min
Lot. No.:	P201104-HS843560	CDL Temp.:	250 °C	B. Conc.:	50%H2O/50%ACN
		Block Temp.:	200 °C		
